# The Effect of Radial Extracorporeal Shock Wave Therapy in the Treatment of Trigger Finger

**DOI:** 10.7759/cureus.8385

**Published:** 2020-06-01

**Authors:** Mahbube Dogru, Mehmet Erduran, Selnur Narin

**Affiliations:** 1 Physiotherapy and Rehabilitation, Institute of Health, Dokuz Eylul University, Izmir, TUR; 2 Orthopaedics and Traumatology, Dokuz Eylul University, Izmir, TUR

**Keywords:** trigger finger, radial extracorporeal shock wave therapy, rehabilitation, hand therapy, prospective cohort

## Abstract

Objective

The aim of this study was to investigate the effect of radial extracorporeal shock wave therapy (rESWT) on the treatment of trigger finger.

Methods

Eighteen patients, who were 2nd grade according to Quinnel classification and diagnosed with trigger finger, were included in this prospective cohort clinical study. The study consisted of only the experimental group and no control group. Eighteen patients with trigger fingers were applied to ten sessions, twice a week, for five weeks of rESWT (2000 impulses, 2 bar, 10 Hz). Pain scores (Numeric Pain Rating Scale), general functional capacity (Quick-DASH), range of motion, grip strength, and pinch strength were evaluated before treatment, after treatment, and three months after the treatment.

Results

Evaluation of ten sessions of rESWT that applied twice a week, for five weeks, was made before treatment, after treatment, and three months after the treatment. Statistical analyses were performed with the Friedman test. As a result of the analyses, there was a decrease in the pain levels (*p *< 0.001) and increase in general functional capacity, grip strength and pinch strength (*p *< 0.001), and range of motion (*p *< 0.001; *p *< 0.005). After the treatment and after three months, all outcome measures showed statistically significant improvements.

Conclusion

rESWT is an effective method to decrease pain severity and improve general functional capacity, range of motion, grip strength, and pinch strength in patients with trigger finger. We concluded that the treatment of rESWT might be a non-invasive option to treat the trigger finger. However, randomized controlled trials are needed to provide more evidence of this treatment

## Introduction

Trigger finger is a stenosing tenosynovitis that is frequently encountered and easy to diagnose which causes pain and disorder in the hand. This occurs as the result of the failure of the interaction between A1 pulley in the level of metacarpal head and the flexor tendon penetrating it. Stenosing tenosynovitis causes the problematic finger to catch and lock during flexion and stay bent (triggering), and it usually occurs after 45 years of age [1]. It is estimated that its prevalence likelihood rate is 3% in the general population and it is in fourth place in line among the reasons why people apply to hand surgery clinics. It is more common in middle-aged women or predisposing factors [2].

Although the etiology of the disease is not yet fully understood, the result of nodular thickening of the tendon and/or narrowing of the tendon sheath (inconsistency between tendon and tendon sheath) leads to restriction of tendon movements. The greatest change is the visible hypertrophy of the pulley itself [3]. The increase in the number of chondrocytes in the A1 pulley of patients with triggered fingers and the fibrocartilage metaplasia are responsible for the pathology [4]. Clinically, tendon compressions at the hands can also lead to stenosis tenosynovitis. Limitation of movement, pain, sensibility, edema, and crepitation may occur. Over time, tendon swells and thickens. As a result, trapping of the tendon in the fibro-osseous canal, limitation of movement, and triggering occur. The tendon becomes nodular at the result of fusiform swelling in the tendon, fibrocartilage metaplasia, or fraying in the tendon [5]. Trigger fingers are usually idiopathic; however, some sources assert that there is a likely correlation with overuse of the hand [6-7]. Also, diabetes mellitus (DM), collagen tissue diseases, rheumatoid arthritis, Dupuytren's disease, amyloidosis, mucopolysaccharide storage disorders, congestive heart failure, genetic predisposition, hypothyroidism, de Quervain's disease, carpal tunnel syndrome, and renal disease are associated with trigger finger [8-9]. Conservative or open and closed surgical loosening is treatment methods. Conservative treatment is recommended in uncomplicated patients who have been referred to shortly after the onset of symptoms. Anti-inflammatory medication, local corticosteroid injection, physiotherapy modalities, and orthosis usage are among the recommended treatments for the trigger finger [10]. Recently, radial extracorporeal shock wave treatment (rESWT) has become a suggestible alternative in treating the muscle-skeletal disorders for patients who fail to respond to the traditional conservative treatment, before moving to the surgical applications [11]. Extracorporeal shock wave therapy (ESWT) is a new orthopedic treatment method based on the principle that high-amplitude sound waves focus on the body's desired region and provide treatment in the body's desired region [12-13]. In rESWT, there is a rocket mechanism used to stimulate pressure waves. The system works with a pneumatically operated pressure generator. The kinetic energy is transmitted to the probe in hand as an elastic shake with rocketing from the compressed air. Throughout the treatment, the prop is in contact with the patient's skin and in this way conveys pressure waves to the skin and subcutaneous tissues of the patient. Pressure waves generated by this mechanism are transmitted thermally [14].

Today, it is possible to see many studies that are carried out to examine the activity of rESWT in many different pathologies such as plantar fasciitis, lateral epicondylitis, Achilles tendinopathies, carpal tunnel, shoulder pathologies; myofascial pain syndrome, spasticity and the number of these studies are gradually increasing [15-17]. There have been numerous studies revealing that rESWT is effective for the other tendon problems like trigger finger. However, there are not many studies carried out directly to see whether rESWT is effective for the trigger finger. As a result; rESWT is an alternative method of treatment that can be performed and treated before surgery, in cases of nonresponsive to conservative treatments, because of the limited number of randomized controlled trials compared with placebo in surgical treatments and the existence studies reporting that rESWT may be effective [11]. The aim of this study is to determine the efficiency of rESWT in the treatment of trigger finger. For this reason, improvement in the general functional capacity, range of motion, grip strength, and pinch strength and decrease in the pain scores were evaluated after treatment and three months after treatment.

## Materials and methods

This study was a prospective cohort study that evaluated before treatment, after treatment, and three months after the treatment. Those who applied to the Orthopaedics and Traumatology Polyclinic, Faculty of Medicine and were diagnosed with the trigger finger were included in the research.

In accordance with the inclusion criterion, each subject in the study was initially informed about the aim, methods to be adapted, and the evaluations to be made both in written form and orally, and each participant was asked to fill out the “Approval Form for Informed Volunteer”.

**Figure 1:**
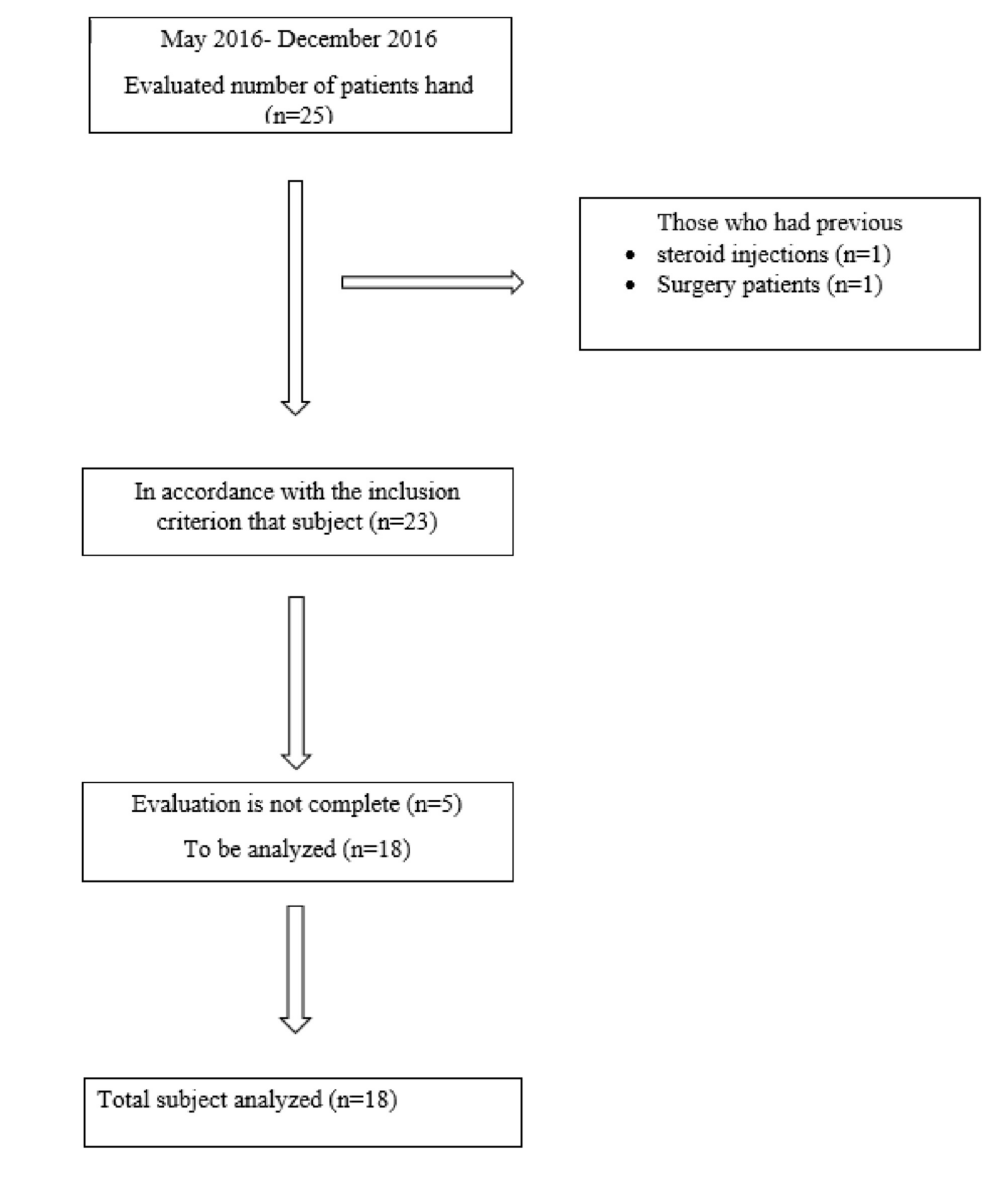
The flow chart of the study

When the power analysis was done prior to the study, the clinically significant change was observed and based upon another study where the change was estimated, with regard to the observable difference of pre- and post-treatment pain grade of patients who were treated with the rESWT with the suitable technique, the effect size was set at 0.8 and when the power to determine this observable difference is 95% (the error level of Type 1) the participants to include in the study was calculated as 18 patients [18]. Figure 1 showed a flowchart of the study.

**Figure 2:**
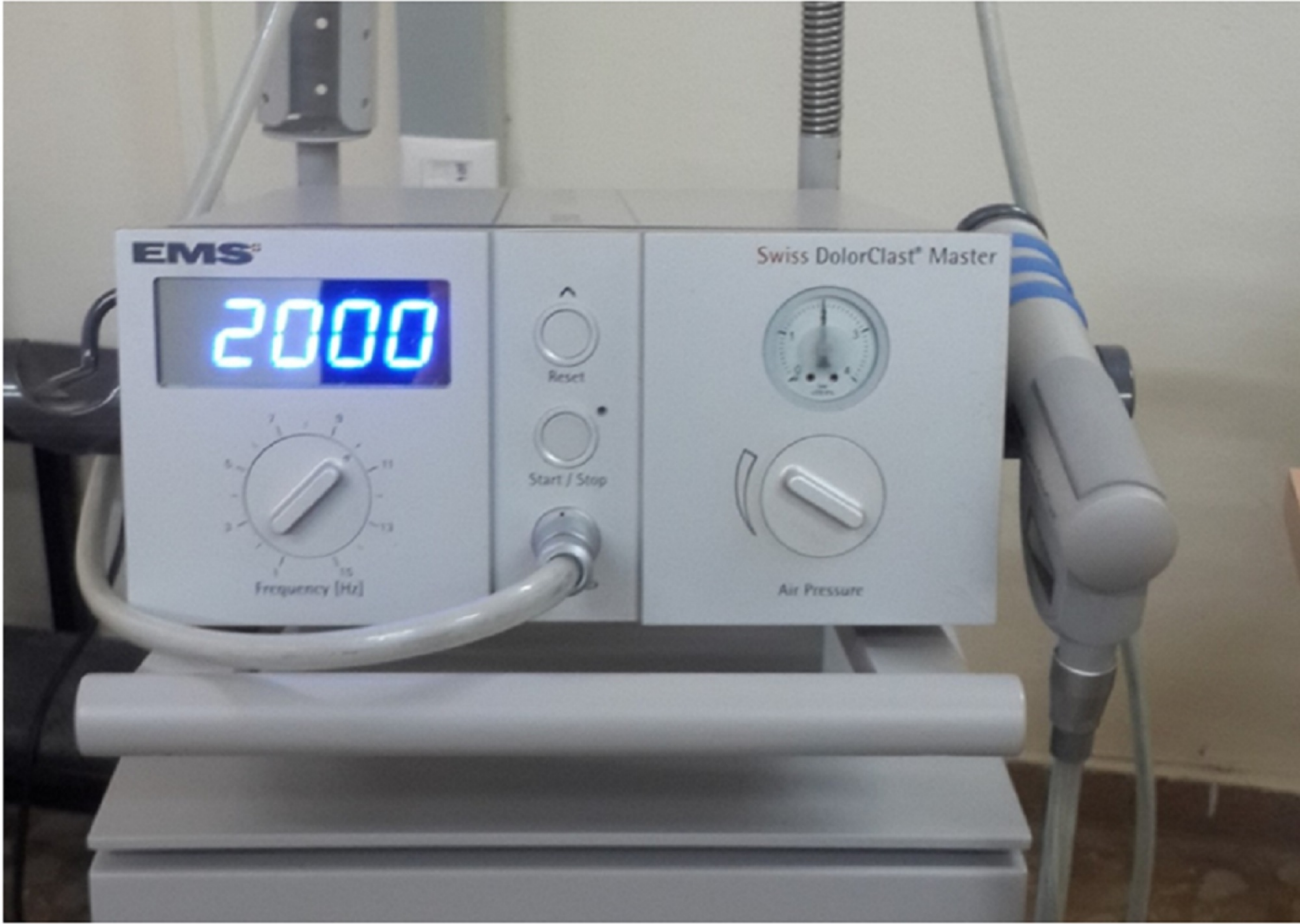
Swiss Dolorclast Master Radial Shock Wave device

Evaluation of five of the 23 patients suitable for the inclusion criteria in the study could not be completed, and hence, these were excluded from the study. The study consisted of only the experimental group and no control group. A Swiss Dolorclast rESWT device (Electro Medical Systems (EMS) S.A Chemin de la Vuarpilliere 31 CH-1260 Nyon, Switzerland) was used for the treatment of trigger finger. Eighteen patients were applied the rESWT (2000 impulses, 2 bar, 10 Hz) 10 sessions, twice a week for five weeks (Figure 2).

**Figure 3:**
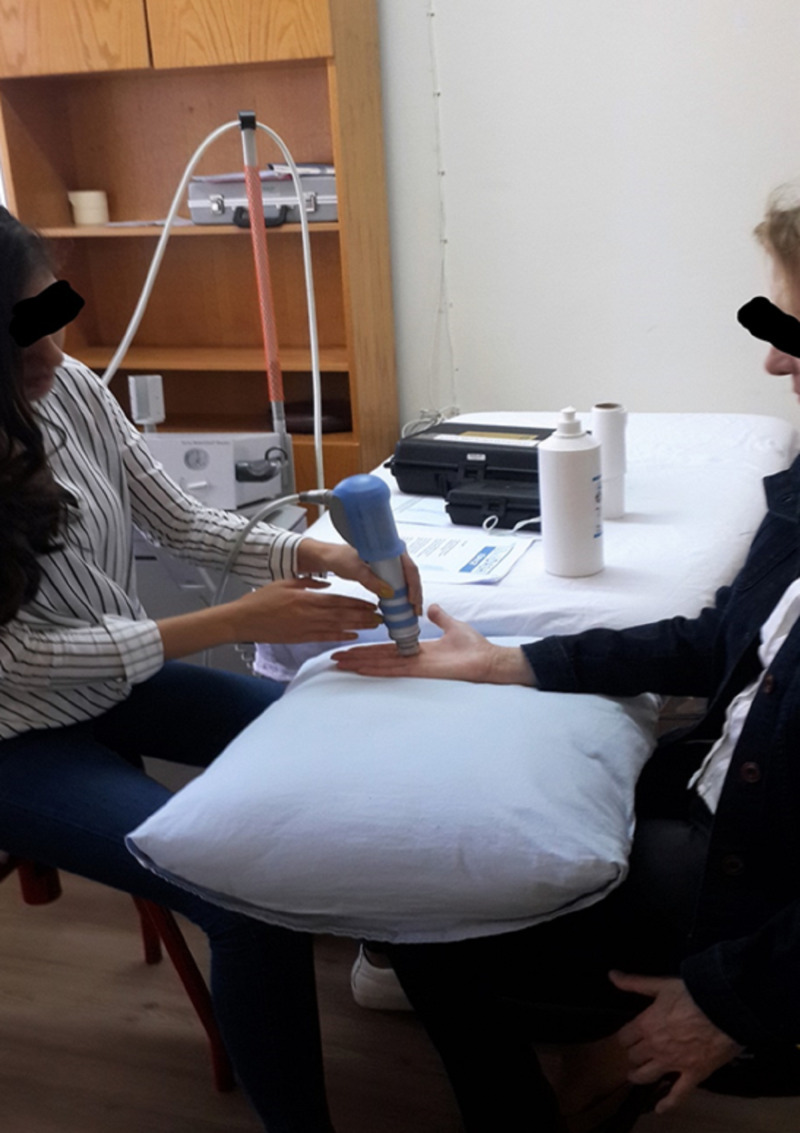
rESWT application

The device pressure was kept low enough to ensure that pain was tolerable but high enough to have a therapeutic effect. The treatment session was identified as ten sessions twice a week. Ultrasound gel was used for the transmission of shockwaves to the patient’s skin. At the end of each session, the patients were suggested to use the finger in daily life activities and no other treatment such as local corticosteroid injection, physiotherapy modalities, orthosis, and surgery was administered during the follow-up period of the study, only they can be using the anti-inflammatory medication. rESWT treatment position was performed as follows: the patients sitting on a chair, with the elbow flexed at 90°, the shoulder adduction beside the body, and the forearm in supination (Figure 3).

Outcome measures

**Figure 4:**
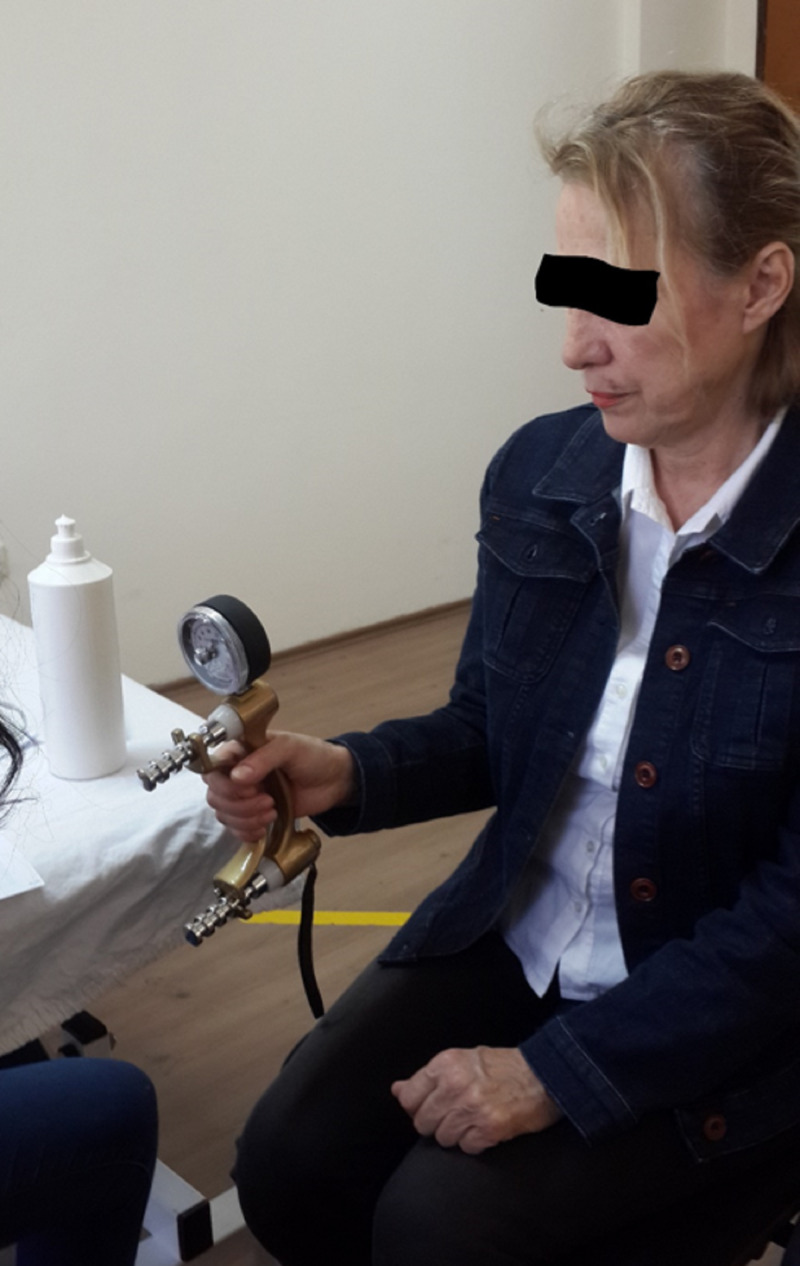
Evaluation of grip strength

All clinical results were evaluated before treatment, after treatment and three months after the treatment. The pain was evaluated using a numeric pain rating scale (NPRS) ranging from 0 (no pain) to 10 (worst possible pain). The general functional capacity with the QuickDASH, the grip strength with the Jamar dynamometer, and the pinch strength with the pinch meter and range of motion with goniometer were evaluated. Position of patients in Jamar hand dynamometer that used to measure grip strength was performed as follows: the patients sitting on a chair, with the elbow flexed at 90°, the shoulder adduction beside the body, and the forearm in semi-pronation. Each patient was asked to squeeze the dynamometer three times with affected hand and for each testing positions. There was a one-minute resting period between each tighten in order to prevent from fatigue. The mean value of the three compressions was taken (Figure 4).

**Figure 5:**
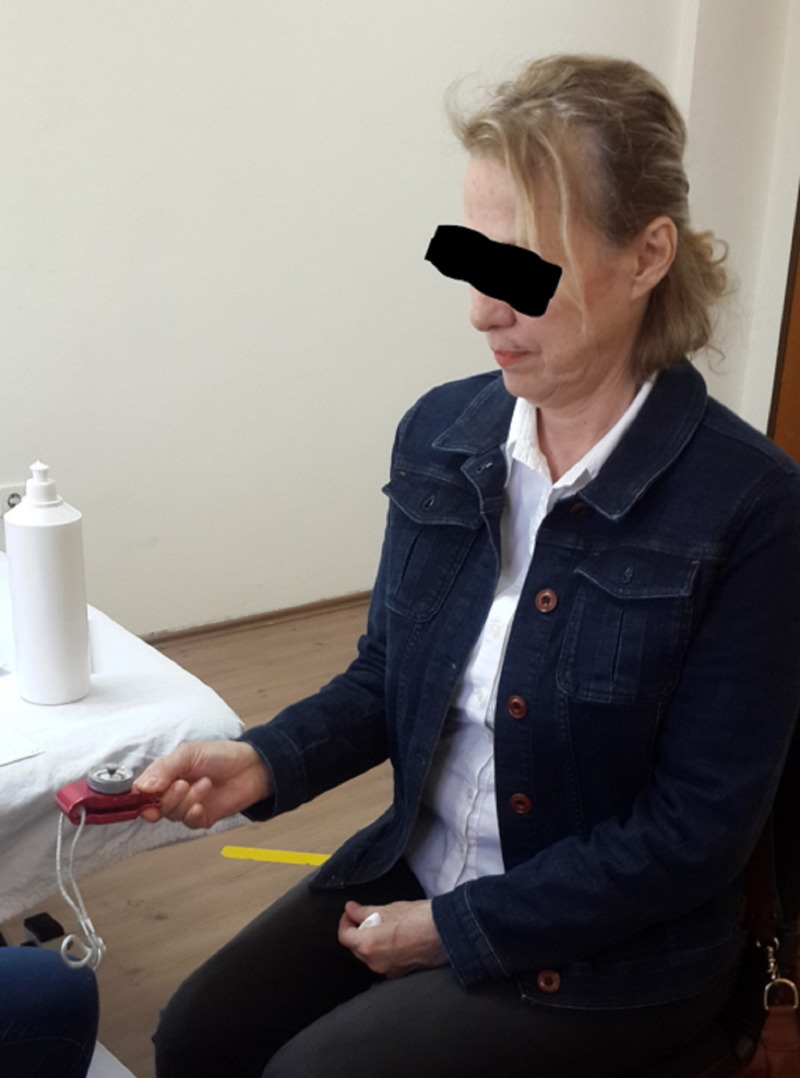
Evaluation of pinch strength

Position of patients in pinch meter that used to measure pinch strength for palmar pinch - thumb, index, and middle fingers - was performed as follows: the patients sitting on a chair, with the elbow flexed at 90°, the shoulder adduction beside the body, and the forearm in semi-pronation. Patients performed one of the pinch compressions above with three successive trials for each affected hand. Patients were given a few minutes rest in between compressions to prevent from fatigue. The mean value of the three compressions was taken (Figure 5).

Inclusion and exclusion criteria of the study

Inclusion Criteria; being older than 18, having the 2. Grade of trigger finger based upon the Quinnell (1980) classification (0st grade, normal finger movements; 1st grade, uneven finger movements 2nd grade, actively correctable triggering; 3rd grade, passive correctable triggering; 4th grade, non-correctable locked finger), no previous surgeries, getting the trigger finger diagnosis by an orthopaedist, being volunteer.

Exclusion Criteria; being diagnosed with rheumatic or systemic inflammatory disease, osteomyelitis, active infection around the hand and the wrist, having anticoagulant treatment for neurological or vascular failure.

 Eighteen patients with trigger fingers were applied to ten-sessions, twice a week, for five weeks of rESWT (2000 impulses, 2 bar, 10 Hz). Evaluation of 10 sessions of rESWT that administered twice a week, for five weeks was made before treatment, after treatment and 3 months after the treatment.

Statistical analysis

The analysis was carried out via “Statistical Package for Social Sciences (SPSS) 22.0 for Windows.” At the end of the analysis, it became clear that the data fell short of meeting the parametric conditions; thus, analysis was done with non-parametric methods. Descriptive statistics were presented as frequency and percentage (%) for categorical variables and median and interquartile range (IQR) according to the distribution of the continuous variables.

In our study, demographic information and information about the disorder of all 18 patients were collected and statistically interpreted prior to the beginning of treatment.). Friedman test was used to compare the measurements of Pain scores (Numeric Pain Rating Scale-NPS), the general functional capacities (Quick-DASH Questionnaire -QD), the range of joint motions, pinch strength (PS) grip strength (GS) of the subjects that were taken before treatment, after treatment and 3 months after treatment. The statistical significance was set as p<0.001 and p<0.005.

## Results

Evaluation of five of the 23 patients who are suitable for the inclusion criteria in the study could not be completed and they were thus excluded from the study. The study was carried out with three men and 15 women, 18 in total who had been diagnosed with the trigger finger. 

In our study, demographics and disease information of patients were obtained and statistically analyzed (Table 1).

**Table 1:** Demographics data of the subjects Data are presented as number and/or median and interquartile range (in parentheses). BMİ, body mass index; n, total evaluated finger number

	rESWT (n=18)
Gender (male/female)	3/15
Dominant hand affected/ non-dominant hand affected	10/8
Affected thump finger / 2nd-5th finger	6/12
Bilateral	2
Age (year)	59.00 (52.00/65.00)
BMI (kg/m2 )	28.52 (23.77/31.22)

The range of joint motions of the affected finger of the 18 patients who were included in the study was measured before treatment, after treatment, and 3 months of treatment. Upon comparing cases with affected thumb in their normal joint motion, a statistically significant result was found (interphalangeal joint flexion (p<0.001); metacarpophalangeal joint flexion (p<0.005; Table 2).

**Table 2:** Comparison of pre-treatment, post-treatment, and 3rd month measurements of the therapeutic group Data are presented as median and interquartile range (in parentheses). IQR, interquartile range; TF-MCF, thumb finger metacarpophalangeal joint flexion; TF-IF, thumb finger interphalangeal joint flexion, MCF, metacarpophalangeal joint flexion; PIF, proximal interphalangeal joint flexion; DIF, distal interphalangeal joint flexion; NPS, numeric pain rating scale; GS, grip strength; PS, pinch strength. QD, Quick-DASH p<0.001, p=0,001, p<0.005

	Pre-median and IQR	Post-median and IQR	3rd-month Median and IQR	P*
TF-MCF	45.00 (40.00/50.00)	45.00 (40.00/50.00)	50.00 (41.25/50.00)	0.004
TF-IF	15.00 (7.50/45.00)	17.50 (7.50/52.50)	20.00 (7.50/60.00)	0.000
2nd-5th finger joint				
MCF	80.00 (80.00/88.75)	82.50 (80.00/90.00)	90.00 (80.00/90.00)	0.000
PIF	100.00 (82.50/110.00)	100.00 (82.50/110.00)	110.00 (92.50/118.75)	0.000
DIF	50.00 (45.00/63.75)	67.50 (51.25/70.00)	70.00 (60.00/75.00)	0.000
NPS	7.00 (5.75/7.00)	2.00 (0.00/3.25)	0.00 (0.00/2.25)	0.000
GS	16.00 (9.50/18.50)	19.00 (15.50/22.50)	18.00 (17.50/25.00)	0.000
PS	3.00 (1.75/4.25)	4.00 (3.00/6.00)	4.00 (3.75/6.00)	0.001
Q-Dash	42.04 (27.26/52.83)	4.54 (0.00/19.31)	0.00 (0.00/9.09)	0.000

During the analysis that was carried out before treatment, after treatment, and 3 months of treatment, when the subjects with 2nd and 5th fingers affected were compared with one another in their normal joint motion, the results were found statistically significant (p<0.001; Table 2).

When the result of the measurements of the pain, the grip strength, the pinch strength, and the general functional capacity of the therapeutic group was compared with the change that occurred in time, it was found that the differences were statistically significant (p<0.01; Table 2).

## Discussion

Our findings show that a ten-session rESWT can be effective on the patients who get grade 2 according to the Quinnell classification in their range of joint motion, the grip strength, the pinch strength, and general functional capacity.

Today, in addition to treating the plantar fasciitis, humeral epicondylitis, calcific rotator cuff tendinitis, union of fracture, late union of fracture, calcified humeral, shock waves are also used in treating the femoral head avascular necrosis, Achilles tendinitis, patellar tendinitis, and osteochondritis dissecans [12,19]. Studies show that rESWT fastens the process of cell regeneration with the neovascularization in the tissue, improving the circulation and cell proliferation, as well and increasing the tissue regeneration in tendon and epicondylitis recovery [20-21]. Although the effect of shock waves on the soft tissue healing process is not definite, it is believed to directly stimulate the healing process and have an effect on neovascularization and decomposition of calcium and also have a neural effect [19]. We think that one of these mechanisms is effective against the thickening of the flexor tendon and its sheath that causes the trigger finger. Although there are differences regarding the dose and the amount of energy to use among studies, the commonly held belief is that high doses cause damage and that they should not be used [22]. As there is no evidence to support the practice of trigger finger in literature and there is no standardized rate to practice rESWT on different body parts, we decided to use 2 bar of pressure force, 10 Hz of frequency and 2000 shots, by taking into consideration the previous studies where rESWT was used on tendinopathy.

There are very little studies in which rESWT was used to treat the trigger finger in literature. The first study using rESWT in trigger finger therapy in literature is the study of N. Malliaropoulos, et al. They retrospectively analyzed 44 patients (49 fingers) treated with the individually tailored rESWT protocol. Triggered graded pain and function were evaluated at baseline and after 1-, 3- and 12 months of treatment. The duration of recurrence and pre-treatment symptom was analyzed. They found significant reductions in pain scores and functional improvement between baseline and all follow-up assessments. At each session, they adjusted 2,000 impulses, 5-6 Hz, 1 to 3 bars and found that rESWT is an effective method of trigger finger treatment. Although there are differences between our study and the study of N. Malliaropoulos et al., based on the number of sessions and he rEWSt protocol, similar results were obtained by both studies upon assessment [23]. These results are favorable for the use of rESWT as an alternative treatment for trigger finger treatment. However, these results are not sufficient for the level of evidence, and hence, more randomized controlled trials are needed.

Another study evaluated the effectiveness of ESWT in the treatment of trigger fingers. Yıldırım P, et al. compared the efficiency of ESWT against the corticosteroid injection in treating trigger finger, and statistically significant findings were obtained from the results of the post-treatment evaluations of both groups. At the end of the study, for patients avoiding steroid injection, it was found that ESWT as a non-invasive method can be used in treating the trigger finger [24]. Significant improvements in the ESWT group suggest that an alternative treatment can be used in trigger finger treatment. Because no patients needed injection or surgery at the end of the third month of our study, rESWT may be recommended as an alternative option for patients avoiding surgery and steroid injection. However, we need more studies to make certain comments, though.

In the evaluation, we made after the study, according to the evaluations that were made before treatment, after treatment and in the 3rd month after it was over, statistically significant decrease in the pain grades of the patients was observed in the rESWT group. That the shock wave treatment is effective against the pain caused by the skeletal muscle disorders is known. The basis for the shock wave treatment has its foundation on the stimulation of soft tissue healing via the inhibition of nociceptors and the decrease of calcification, neovascularization, and hyperemia [25]. It is also believed to have a direct effect on the nociceptors and hyperstimulation that block the gate control mechanism [19]. It is present in the literature that in other disorders where rESWT is used, it has a positive effect on the pain grade. In the systematic review where the effect of the shock waves in treating the soft tissue problems by Cathy Speed was recorded, it was stated that the pain level was decreased via the use of rESWT on the soft tissue problems and especially on the plantar fasciopathy cases [26]. In a study by Helbig K et al. 150 cases with plantar fasciitis and lateral and medial epicondylitis were practiced with shock wave treatment, and as a result, some good and very good progress was made: improvements were observed in patients with plantar fasciitis (80%), epicondylitis (78%), and medial epicondylitis (58%). Patients with a diagnosis of this order were recommended to prefer ESWT for treatment [27]. A study evaluated the effectiveness of ESWT on pain and triggering in the treatment of trigger fingers. In the results of the study, it was found that symptoms completely resolved in the 30 of 32 patients. According to the results of the study, ESWT can be an effective therapy for trigger fingers in 2. and 3. The severity grade may be an alternative for a non-invasive method such as steroid injection [28]. Another study found similar results in pain on the effectiveness of ESWT in trigger finger treatment [29].

In our study, we also evaluated grip strength and pinch strength. Due to problems such as avoiding the use of affected hands, the grip strength and pinch strength of the trigger finger patients can be reduced. As a result of the evaluations, we found statistically significant improvements in these parameters. Gündüz et al. compared the activities of physiotherapy (hot pack, ultrasound, and friction massage), local steroid injection and ESWT on pain levels, grip strength, and pinch strength of 59 patients. In the end, what was found out was that physiotherapy, steroid injection, ESWT had positive effects on the pain levels and the grip strength in treating the earlier stages of lateral epicondylitis and that the ESWT had a long-term effect on grip strength [30]. This study, which was practiced on the lateral epicondylitis patients with tendinopathy diagnosis like trigger finger, shows that the ESWT is useful in treating the tendinopathies; additionally, it supports the result of our study in that the ESWT is also effective in improving the grip strength and decreasing the pain grade. There are no previous studies demonstrating the positive effects of rESWT on the grip strength and the pinch strength in the treatment of trigger fingers and there is a need for more studies to better discuss these results, which are the first evidence.

Range of joint motions can be reduced in the affected finger due to reasons such as the frequency of triggering, severity, avoiding pain or prevent it from getting worse. In our study, we evaluated in our study, we evaluated a range of joint motions with a goniometer. There was a statistically significant increase in the range of joint motions. Therefore, we thought especially that improvement in pain, which is one of the major causes of a decrease in joint movement, might cause an increase in the range of joint motions.

When we examined the results of 18 patients after rESWT treatment in our study, its efficiency on the pain levels, the pinch strength, the grip strength, the range of joint mobility, and the general functional capacity were found to be significant.

Strengths of the study

rESWT is a newer method. Despite this, its use has been increasing in recent years. With the increase in usage rate, it is possible to see many studies in the literature to investigate the effectiveness of RESWT in many different pathologies such as heel spur, lateral epicondylitis, Achilles tendinopathies, shoulder pathologies, myofascial disorders, and spasticity. But there are very few studies in the literature to evaluate the effectiveness of rESWT for the treatment of trigger finger. For this reason, our work is an important issue for the literature. All patients completed the three-month follow-up. The high efficacy of rESWT demonstrated in this study is similar to the results of previous studies on rESWT for tendinopathy. All patients completed the study, but only five patients who suitable inclusion criteria were not evaluated because they did not complete the treatment.

Limitation of the study

As this study is a prospective cohort study, it has some limitations. There was no randomization and there was no control group in this study. Although the biggest limitation of the study is believed that there was no control group, as we plan to make use of different control groups in the upcoming studies, we meant to evaluate the result of this study without including any control groups.

## Conclusions

The study provides evidence for the effectiveness of rESWT for the treatment of trigger finger. The fact that there were improvements in the pain levels and the general functional capacity of the subjects who were evaluated before treatment, after treatment, and in the third month supports that the efficiency of the treatment has promising results. Randomized controlled trials are required to prove well the evidence of the rESWT effect.
